# Differences among specialists in using the Electronic Medical Record alert system for HCV screening in outpatients at a large general hospital

**DOI:** 10.1371/journal.pone.0333940

**Published:** 2025-10-10

**Authors:** Cheng-Kuan Lin, Yu-Sen Peng, Chi-Yu Yang

**Affiliations:** 1 Department of Internal Medicine, Far Eastern Memorial Hospital, Banqiao, Taiwan; 2 Deputy Superintendent, Far Eastern Memorial Hospital, Banqiao, Taiwan; 3 Cardiovascular Center, Far Eastern Memorial Hospital, Banqiao, Taiwan; Kaohsiung Medical University, TAIWAN

## Abstract

**Background & objectives:**

Some hepatitis C virus (HCV) patients remain underdiagnosed at a large hospital. The electronic medical record (EMR) alert system can increase in-hospital screening. This study evaluates the effectiveness of screening among outpatients and assesses variations in physician engagement across different specialties.

**Methods:**

From December 2022 to April 2023, outpatients aged 45–79 years, with diabetes mellitus or chronic kidney disease, who did not have anti-HCV data in their EMR were identified. A pop-up alert prompted physicians to either order anti-HCV testing or refer patients for a health check-up. The frequency of alerts, physician response rates, blood test completion rates, and HCV seropositivity were analyzed by specialty.

**Results:**

Of 85,320 alerts generated, 61.4% elicited a physician response. Internal Medicine clinics had significantly higher response rates than non-Internal Medicine clinics (77.5% versus 44.6%, P < 0.01). Among patients with physician responses, 30.9% completed the study, with markedly higher completion when blood tests were directly ordered compared to referrals (88.4% versus 7.2%, P < 0.01). The monthly number of screenings increased tenfold after the system was implemented. Overall, 22,377 patients (26.2%) were screened, and 485 (2.17%) were seropositive. One-fourth of seropositive cases were identified in non-Internal Medicine clinics, where the positivity rate was higher (2.7% versus 2.04%, P = 0.01).

**Conclusions:**

Short-term use of the EMR alert system increased HCV detection among outpatients. The default test orders in the EMR improved completion rates more than referrals to a health check-up. There were differences in physician engagement and seropositive case detection among specialties. Targeted interventions, particularly among specialties with lower engagement, are necessary to improve the success of HCV screening.

## Introduction

Since the introduction of direct-acting antiviral (DAA) agents, which achieve high sustained virological response (SVR) rates, the elimination of the hepatitis C virus (HCV) has become a feasible goal. In 2016, the World Health Organization (WHO) set a target to eliminate viral hepatitis by 2030 [1]. Taiwan accelerated its HCV elimination efforts since 2018, aiming to treat 80% of eligible individuals by 2025 [2]. The major barriers are low awareness of infection and inadequate linkage to care. Globally, the majority of individuals with HCV are unaware of their infection, with fewer than 25% having been diagnosed [3]. A United States-based survey found that fewer than 50% of individuals were aware of viral hepatitis [4,5]. In Taiwan, before the implementation of universal screening, HCV awareness rates ranged from 36.2% to 66.5% [6,7]. Expanding HCV screening, particularly in high-prevalence populations, is important for achieving the target of elimination. In the United States, previous guidelines recommended screening individuals born between 1945 and 1965. Since 2020, guidelines have been updated to recommend one-time screening for all adults aged 18–79 years to improve diagnosis and treatment rates [8]. Japan launches a national screening program targeting individuals aged 40 and older. Similarly, Taiwan includes anti-HCV and HBsAg testing in its complementary national health check-up for individuals aged 45–79 years [9]. In our hospital, HCV screening is routinely performed for high-risk groups, including patients undergoing hemodialysis, intravenous drug abusers receiving methadone maintenance therapy, individuals with human immunodeficiency virus infection, and patients being evaluated for chemotherapy. For staff safety, screening is also conducted before surgical procedures. However, a proportion of patients remain unscreened, and the exact rate of unawareness within the hospital setting is unknown.

Electronic medical record (EMR) alert systems have been shown to improve HCV screening rates in various settings, such as primary care clinics and emergency departments, particularly for birth cohort screening [10–12]. The addition of automated testing features may further enhance screening uptake [13]. However, there is limited evidence on the use of EMR alerts for universal HCV screening in large general hospitals, and little is known about the uptake of screening across different medical specialties.

In this study, we implemented an extended HCV screening program designed to identify outpatients without an anti-HCV antibody record in their EMRs. A pop-up alert prompted physicians to either order the screening test directly or refer patients to a health check-up for testing. We analyzed alert frequency, physician responses, and test completion rates across two screening approaches. The identified HCV cases were compared by medical specialty and age group.

## Materials and methods

### Hospital setting and study design

This study was conducted at Far Eastern Memorial Hospital, a 1,300-bed tertiary medical center located in northern Taiwan, with approximately 7,000 outpatient visits per day. To support national efforts toward HCV elimination through in-hospital screening, the hospital implemented an extended HCV screening program using an EMR alert system. Before the program’s implementation, multidisciplinary meetings were held with key stakeholders, including senior hospital administrators and directors of the Departments of Hepato-Gastroenterology, Infectious Diseases, Laboratory Medicine, and Information Technology (IT). Program details were communicated to hospital staff via the internal electronic bulletin system. Educational sessions covering HCV screening and treatment were conducted during faculty meetings and resident didactics, especially in departments managing a higher burden of HCV, such as Internal Medicine, Family Medicine, and the Cardiovascular Center. The program was monitored biweekly by the administrative supervisory committee. The goal of the alert system was to ensure that more than 90% of visiting outpatients had a documented awareness of or diagnosis of their HCV status.

Before the intervention, HCV screening was performed during routine blood testing or periodic health check-ups. From December 2022 to April 2023, an EMR-based extended HCV screening program was conducted in the outpatient departments. The eligible patients included those aged 45–79 years, with diabetes mellitus (DM) or chronic kidney disease (CKD) who lacked anti-HCV antibody data in their EMRs. Exclusion criteria included visits to pediatric clinics, self-pay clinics, examination rooms, traditional medicine services, and nursing home services. The extended screening program targeted individuals aged 45–79 years who are eligible under the government’s criteria for free HBsAg and anti-HCV testing. For patients with positive anti-HCV results, reflex HCV RNA testing was reimbursed. Screening also included individuals with DM or CKD, who are known to have higher HCV prevalence. In Taiwan, DM patients are mostly enrolled in a diabetes management program if they attend more than two visits within three months. Similarly, CKD patients are enrolled in either the early CKD or pre-end-stage renal disease (pre-ESRD) programs based on disease stage and follow-up frequency.

### The working of the EMR alert system for HCV screening

**Fig 1:**
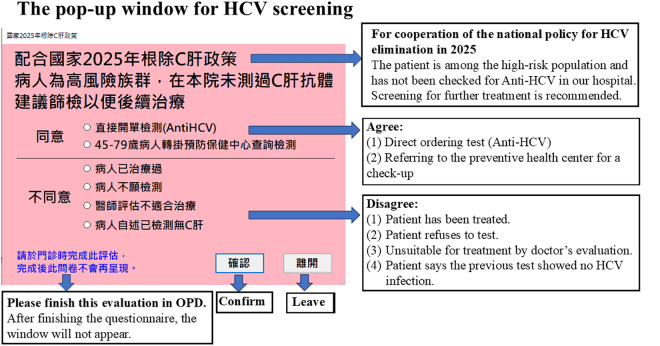
Pop-up alert window in the EMR system used at Far Eastern Memorial Hospital to promote intra-hospital HCV screening among outpatients.

When eligible patients visited outpatient clinics, a pop-up alert (Fig 1) appeared in the EMR, prompting physicians to offer HCV testing to them. Physicians could choose one of the following options:

1. Agree to a check-up: either by directly ordering an anti-HCV test or by referring the patient to the health check-up center for hepatitis B and C screening.
2. Disagree to check-up: with reasons including prior treatment, patient refusal, medical unsuitability for therapy, or patient-reported prior testing with no HCV infection.

If the patient accepted testing, the anti-HCV antibody test was automatically ordered. For patients preferring health check-up screening, a referral sheet was printed for presentation at the health check-up center. If the physician did not respond to the alert, it would reappear at subsequent visits until a selection was made, which was then recorded in the EMR.

### Data collection and analysis

Monthly data were collected on the number of alerts triggered and physician responses, categorized by specialty. Physicians were grouped into two categories:

- Group I (Internal Medicine Clinics): including Hepato-Gastroenterology, Endocrinology, Nephrology, Chest Medicine, Cardiology, Oncology, Infectious Disease, Rheumatology, General/Internal Medicine, Integrated Medicine, Family Medicine, and Neurology.
- Group II (Non-Internal Medicine Clinics): including General Surgery, Neurosurgery, Plastic Surgery, Cardiovascular Surgery, Thoracic Surgery, Proctology, Trauma, Urology, Orthopedics, Gynecology, Otorhinolaryngology, Ophthalmology, Dentistry, Anesthesiology, Dermatology, Psychiatry, Occupational Medicine, Radiation Therapy, and Hyperbaric Oxygen Therapy.

The study was observational and retrospective, analyzing alert frequency, physician response behavior, test completion rates, and HCV seropositivity outcomes across different specialty departments. We compared completion rates between patients screened via direct test orders and those referred to health check-ups. The number and seropositivity rate of identified HCV cases were analyzed by specialty and age group. A dedicated hepatitis case manager conducted the patient’s follow-up and coordinated their care. Finally, we documented the whole cascade of care from anti-HCV testing to initiation of DAA therapy.

This study was conducted in accordance with the Helsinki Declaration of 1975, as revised in 2008, and approved by the Institutional Review Board of Far Eastern Memorial Hospital (FEMH-112215-E) for studies involving human subjects. Written informed consent for DAA therapy was obtained from all participants.

### Statistical analysis

Continuous variables are presented as means ± standard deviations and ranges. Categorical variables are reported as counts and percentages. Differences in continuous variables were analyzed using the Mann–Whitney U test, and categorical variables were compared using the Chi-square test. All statistical tests were two-tailed, and a P-value < 0.05 was considered statistically significant. Analyses were performed using Stata software (version 13.0; Stata Corp, College Station, TX, USA).

## Results

### Alerts in outpatients and physician responses

A total of 85,320 alerts (19%) were triggered among 450,129 outpatient visits. A total of 66,554 patients were enrolled, receiving an average of 1.3 alerts from the system. The mean age was 61.6 ± 9.5 years, and 44.5% were male. The program included 8,482 patients (12.7%) in the diabetes care program and 994 patients (1.5%) in the early CKD/pre-ESRD program.

**Fig 2:**
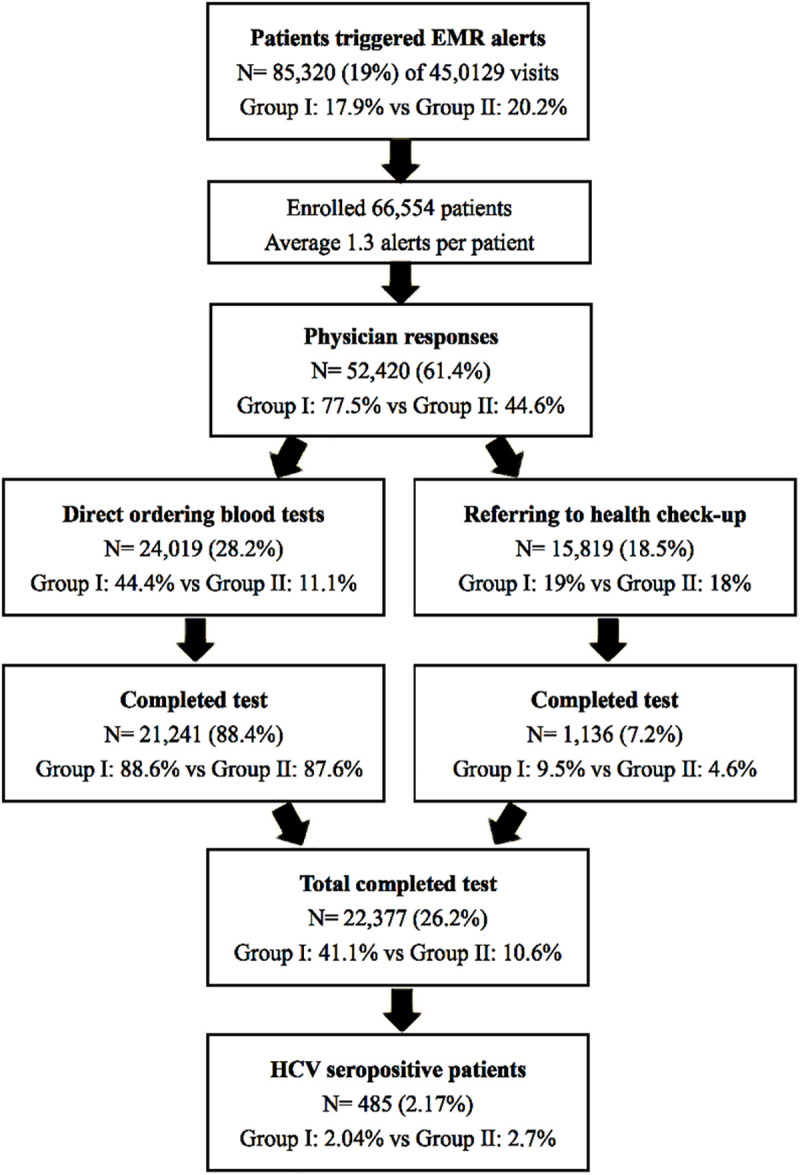
The flowchart illustrating the study results. Group I: Internal Medicine clinics; Group II: Non-Internal Medicine clinics.

Group I accounted for 43,715 alerts (17.9%) across 244,511 visits, while Group II generated 41,605 alerts (20.2%) across 205,618 visits. The proportion of patients without anti-HCV data who received an alert among all screened patients was significantly higher in Group II than in Group I (P < 0.01) (Fig 2). The departments with the highest percentages of patients lacking anti-HCV data included Cardiovascular Surgery (33.3%), Cardiology (28.8%), Ophthalmology (28.3%), Neurology (27.7%), and Neurosurgery (27.4%) (Table 1).

**Table 1:** Top 10 departments in physicians’ responses to EMR alerts.

Rank	Patients without anti-HCV (%)	Physician answered (%)	Left without answering (%)	Direct blood test order (%)	Referral to health check-up (%)
#1	CVS (33.3%)	Occup (100%)	Oph (78.8%)	Meta (83.3%)	Psychi(70.7%)
#2	CV (28.8%)	IM (100%)	Anes (76.5%)	Onco (63.2%)	RT (56.0%)
#3	Oph (28.3%)	Proct (96.0%)	GYN (70.6%)	Nephro (62.7%)	Proct (43.9%)
#4	Neuro(27.7%)	ID (94.5%)	Orth (70.6%)	GM (51.6%)	NS (41.3%)
#5	NS (27.4%)	Onco (93.6%)	PMR (64.0%)	GI (40.6%)	Trauma(37.6%)
#6	Orth (26.6%)	Psychi(92.6%)	Dent (63.5%)	CV (40.0%)	Plastic (30.6%)
#7	Meta(25.5%)	GI (92.5%)	Uro (57.7%)	CVS (39.8%)	GS (29.0%)
#8	Uro (24.7%)	Meta (89.8%)	Derma(50.6%)	Rheuma(36.8%)	CV (28.3%)
#9	Occup(24.4%)	CS (81.5%)	GS (50.5%)	Occup (36.4%)	Chest (27.4%)
#10	ENT (18.6%)	Nephro(80.8%)	ENT (47.7%)	Neuro(34.3%)	ENT (25.8%)
Average	19.0%	61.4%	38.6%	28.2%	18.5%

Abbreviations: Chest, Chest Medicine; GM, General/Internal Medicine; Neuro, Neurology; Oph, Ophthalmology; Plastic, Plastic Surgery; Orth, Orthopedics; HyperO, Hyperbaric Oxygen Center; PMR, Physical Medicine and Rehabilitation; Meta, Endocrinology; Onco, Oncology; IM, Integrated Medicine; GYN, Gynecology; Dent, Dentistry; CS, Chest Surgery; Anes, Anesthesiology; GI, Hepato-Gastroenterology; Rheuma, Rheumatology; Derma, Dermatology; GS, General Surgery; Uro, Urology; Psychi, Psychiatry; RT, Radiation Oncology; Nephro, Nephrology; ID, Infectious Disease; CV, Cardiology; ENT, Otorhinolaryngology; NS, Neurosurgery; Proct, Proctology; Occup, Occupational Medicine; CVS, Cardiovascular Surgery.

Over the 5 months, physicians responded to 61.4% of alerts. Group I physicians responded significantly more often than Group II (77.5% vs. 44.6%, P < 0.01). Departments with the highest physician response rates included Occupational Medicine (100%), Integrated Medicine (100%), Proctology (96%), Infectious Disease (94.5%), Psychiatry (92.6%), and Oncology (93.6%) (Table 1).

Among patients who declined screening, 204 (0.2%) reported having completed prior therapy, 7,852 (9.2%) refused testing, 706 (0.8%) were deemed unsuitable for treatment, and 3,754 (4.4%) reported having undergone prior testing elsewhere with no HCV infection. Refusals were more common in Group II than in Group I (11.7% vs. 6.9%, P < 0.01). Group I had a higher proportion of patients reporting known negative anti-HCV status (6.1% vs. 2.6%, P < 0.01).

**Fig 3:**
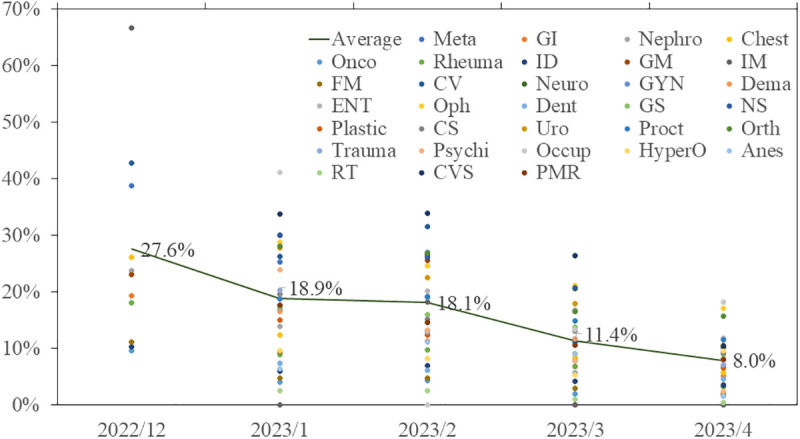
Monthly trend in the proportion of EMR alerts for HCV screening among visiting outpatients. Abbreviations: Chest, Chest Medicine; GM, General/Internal Medicine; Neuro, Neurology; Oph, Ophthalmology; Plastic, Plastic Surgery; Ortho, Orthopedist Surgery; HyperO, Hyperbaric Oxygen Center; PMR, Physical Medicine and Rehabilitation; Meta, Endocrinology; Onco, Oncology; IM, Integrated Medicine; GYN, Gynecology; Dent, Dentistry; CS, Chest Surgery; Trauma, Trauma Surgery; Anes, Anesthesiology; GI, Hepato-Gastroenterology; Rheuma, Rheumatology; FM, Family Medicine; Derm, Dermatology; GS, General Surgery; Uro, Urology; Psychi, Psychiatry; RT, Radiation Oncology; Nephro, Nephrology; ID, Infectious Disease; CV, Cardiology; ENT, Otorhinolaryngology; NS, Neurosurgery; Proct, Proctology; Occup, Occupation Medicine; CVS, Cardiovascular Surgery.

The proportion of outpatients needing alerts declined significantly from 27.6% to 8.0% over the study period (P < 0.01) (Fig 3). However, more than 10% of patients in departments such as Occupational Medicine (18.2%), Ophthalmology (17.1%), Oral Surgery (13.3%), Otorhinolaryngology (11.8%), Proctology (11.5%), Gynecology (10.9%), Urology (10.6%), Cardiovascular Surgery (10.4%), Neurology (10.3%), and Neurosurgery (10.2%) still lacked HCV data. The finding indicated a need for continued alerts in these departments.

### Methods of screening and completion rates

Among patients offered screening, 24,019 (28.2%) underwent direct blood test orders, and 15,819 (18.5%) were referred for hepatitis screening via health check-ups. Group I physicians more frequently used direct test orders than those in Group II (44.4% versus 11.1%, P < 0.01), while referral rates were similar between groups (19.0% versus 18.0%, P = 0.11) (Fig 2).

Departments with the highest use of direct test orders were Endocrinology (83.3%), Oncology (63.2%), Nephrology (62.7%), General/Internal Medicine (51.6%), and Hepato-Gastroenterology (40.6%). Departments with the highest use of referrals were Psychiatry (70.7%), Radiation Oncology (56.4%), Proctology (43.9%), Neurosurgery (41.3%), and Trauma (37.6%) (Table 1).

A total of 22,377 patients completed blood testing. The monthly number of HCV screenings increased tenfold after implementing the EMR alert system (from 446 to 4,475 per month). The completion rate was significantly higher with direct orders compared to referrals (88.4% versus 7.2%, P < 0.01). Completion rates were also higher in Group I than in Group II (41.1% versus 10.6%, P < 0.01). Among patients receiving direct orders, completion rates were similar between the two groups (88.6% versus 87.6%, P = 0.06). In contrast, referral-based completion was higher in Group I (9.5% versus 4.6%, P < 0.01) (Table 2).

**Table 2:** Completion of blood test and HCV seropositivity rate by screening method and specialty group.

Method A: Direct ordering of blood tests				
	Total (N = 24,019)	Group I (N = 19,386)	Group II (N = 4,633)	P value
Completion test	88.4%	88.6%	87.6%	0.06
Anti-HCV(+) rate	2.16%	2.04%	2.64%	0.02*
**Method B: Referring to a health check-up**				
	**Total (N = 15,819)**	**Group I (N = 8,323)**	**Group II (N = 7,494)**	
Completion test	7.2%	9.5%	4.6%	<0.01*
Anti-HCV(+) rate	2.4%	1.9%	3.5%	<0.01*
**All methods require the completion of blood tests.**				
	**Total (N = 22,377)**	**Group I (N = 17,937)**	**Group II (N = 4,404)**	**P value**
Age (mean ± SD)	63.3 ± 10.1	63.3 ± 10.3	63.3 ± 9.2	0.96
Sex (male)	52.7%	51.2%	63.3%	<0.01*
DM	30.3%	33.9%	5.0%	<0.01*
CKD	3.3%	3.6%	0.8%	<0.01*
Anti-HCV(+) rate	2.17%	2.04%	2.7%	0.01*

Group I: Internal Medicine clinics. Group II: non-Internal Medicine clinics. “All methods” refers to the total number of patients screened in each department, regardless of the specific screening method used. *P < 0.05 indicates statistical significance.

### Characteristics of HCV seropositive patients

Among the 22,377 patients who completed blood testing, 485 (2.17%) were seropositive for HCV antibodies. The seropositivity rate was higher in females than in males (2.51% versus 1.68%, P < 0.01). However, HCV seropositivity did not differ significantly between patients with and without DM (2.3% versus 2.1%, P = 0.62). Similarly, there was no difference between patients with and without CKD (3.0% versus 2.6%, P = 0.25).

Group I consisted of 366 (75.5%) seropositive patients, and Group II consisted of 119 (24.5%) seropositive patients. The HCV seropositivity rate was higher in Group II than in Group I (2.7% versus 2.04%, P = 0.01) (Table 2), suggesting the importance of HCV screening beyond high-risk departments. The departments with the highest case numbers were Cardiology (n = 112), Endocrinology (n = 100), Hepato-Gastroenterology (n = 64), Urology (n = 32), and Neurology (n = 31). Departments with the highest seropositivity rates included Psychiatry (21.3%), Trauma (13.3%), Neurosurgery (4.2%), Chest Medicine (4.1%), and Dermatology (3.9%) (Table 3).

**Fig 4:**
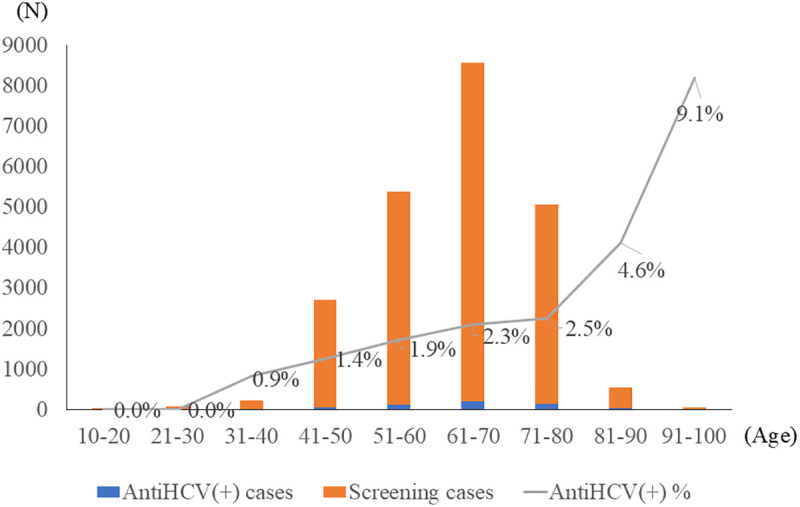
Distribution of HCV seropositive cases and corresponding seropositive rates across different age groups.

The mean age of seropositive patients was 65.8 ± 9.9 years (range, 38–98), with 56.5% of the patients being female. Seropositivity increased with age (Fig 4). Among these patients, 26.2% had diabetes, and 2.9% had CKD. In the year preceding screening, these patients had a mean of 7.8 ± 7 outpatient visits. They consulted 2.3 ± 1.9 different specialties, suggesting that frequent healthcare use did not guarantee a prior diagnosis of HCV in the absence of EMR alerts.

**Table 3:** Top ten departments by number of HCV seropositive cases and seropositivity rate.

Rank	Top departments by case Number (Anti-HCV positive cases)	Cases (n)	Top departments by seropositive rate	Rate (%)
#1	CV	112	Psychi	21.3%
#2	Meta	100	Trauma	13.3%
#3	GI	64	NS	4.2%
#4	Uro	32	Chest	4.1%
#5	Neuro	31	Derm	3.9%
#6	Psychi	23	RT	3.8%
#7	CVS	17	ID	3.6%
#8	Nephro	16	GI	3.6%
#9	Chest	12	GS	2.9%
#10	Onco	11	Uro	2.8%
Total		485		2.17%

Departments are ranked by either the absolute number of HCV antibody-positive cases or the highest seropositivity rate among patients tested.

Abbreviations: CV, Cardiology; Meta, Endocrinology; GI, Hepato-Gastroenterology; Uro, Urology; Neuro, Neurology; Psychi, Psychiatry; CVS, Cardiovascular Surgery; Nephro, Nephrology; Chest, Chest Medicine; Onco, Oncology; Trauma, Trauma Surgery; NS, Neurosurgery; Derm, Dermatology; RT, Radiation Oncology; ID, Infectious Disease; GS, General Surgery.

### Cascade from screening to treatment

Among the 485 seropositive patients, 310 (63.9%) underwent HCV RNA testing, and 116 (37.4%) were viremic. Of the remaining 175 untested patients, reasons for not undergoing RNA testing included death (n = 10), prior interferon-based treatment (n = 18), treatment at other hospitals (n = 69), and loss to follow-up (n = 79).

Among the 116 viremic patients, only 44 (37.9%) had elevated serum transaminase levels, and 11 (9%) had liver cirrhosis. Finally, 103 patients (88.8%) initiated DAA therapy, resulting in an SVR (intention-to-treat) of 93.2%.

## Discussion

With the advent of DAA therapy, HCV infection has become a curable disease. The WHO set a target to eliminate viral hepatitis as a public health threat. However, global awareness of HCV remains suboptimal, with only 33% (range 29–65%) of infected individuals estimated to be aware of their status by 2020 [3]. Identifying the large pool of undiagnosed individuals is essential to achieving elimination. Several countries have expanded their HCV screening policies to promote the goal [8,9].

Our findings demonstrated that 27.6% of outpatients still lacked anti-HCV data in their EMRs before the implementation of the alert system. After five months of this intervention, the proportion decreased to 8.0%, reflecting the system’s potential to scale up HCV screening in a large general hospital rapidly. If physicians respond appropriately and patients complete testing, such an approach could support the WHO’s goal of achieving more than 90% diagnostic coverage.

Before the implementation of universal screening, HCV testing in our hospital was primarily limited to Internal Medicine clinics serving high-risk populations or patient-initiated health check-ups. As expected, we found a significantly higher proportion of patients without anti-HCV data in non-Internal Medicine clinics, reflecting the importance of institution-wide strategies that involve all specialties.

Previous studies have shown that EMR alert systems can significantly increase HCV screening rates, particularly among baby boomers [14–19]. Reports indicate increases of 5- to 15-fold in screening uptake, with noted improvement from 28% to 72% within the first year [10,11,20]. A randomized controlled trial found that EMR alerting increased HCV case identification fivefold compared with standard care [21]. Meta-analyses and cost-effectiveness studies also support EMR-based alerting as an efficient strategy to improve screening and case detection at a relatively low cost [22–24].

EMR alerts have been effectively deployed not only in emergency departments and among high-risk populations, such as patients with DM, CKD, or cancer, but also in broader outpatient settings [12,25–28]. Our intervention led to a 10-fold increase in the number of individuals screened, and the screening rate rose from 19.1% to 53% within five months.

Nonetheless, reaching the > 90% screening target remains challenging. Even with EMR-based alerts, physician engagement varied significantly by specialty. Alerts were often ignored or dismissed, particularly by non-Internal Medicine specialists. In our study, only 30.9% of patients eventually received appropriate screening or documentation. The test completion rate was higher in Internal Medicine clinics. This disparity is consistent with prior studies in patients undergoing screening before endoscopic procedures, as well as those with DM or CKD [26,27,29].

Several factors may explain these specialty-based differences: (1) Internal Medicine patients tend to have more frequent, scheduled follow-ups; (2) Internal Medicine physicians are typically more experienced in HCV care; (3) non-Internal Medicine physicians often focus on patient’s primary complaints; and (4) differing disease patterns and patient expectations may also affect perceived relevance of screening. Strategies to enhance screening uptake include launching targeted educational programs for both clinicians and patients, introducing performance incentives, and integrating rapid point-of-care testing.

We found that test completion rates were substantially higher for default-ordered tests compared to those requiring referral to health check-up services (88.4% versus 7.2%, P < 0.01). Possible test completion barriers, such as the distance between clinics, health check-up centers, and laboratories, as well as long wait times, likely contribute to this discrepancy. Therefore, embedding default orders into the EMR alert system is recommended to optimize the completion of screenings [30].

Despite the intervention’s success, physician responsiveness to alerts varied by specialty. If the alert was dismissed and the patient did not return, a screening opportunity was missed. Frequent alerts can lead to alert fatigue and influence physician responsiveness. The clinician’s responsiveness has been shown to decline by 30% with each additional alert during a patient encounter [31]. To minimize fatigue, the frequency and timing of pop-up alerts should be adjusted when the screening goal is achieved.

In Taiwan, the estimated anti-HCV seroprevalence is 3.3%, with regional variation. The northern region, where our hospital is located, has a prevalence of approximately 2% [2,32]. Our study’s seropositive rate of 2.17% was approximated with these estimates. Departments with high outpatient volumes, such as Cardiology, Endocrinology, Hepato-gastroenterology, Urology, and Neurology, accounted for the majority of seropositive cases. The highest seropositive rate in the Department of Psychiatry likely reflects a higher prevalence of substance use disorders in screening subjects. The departments with the most significant burden of undiagnosed HCV highlight potential targets for enhanced screening interventions.

Our data confirmed that HCV seroprevalence increases with age, supporting the current Taiwanese screening policy for individuals aged 45 years and above. The HCV seropositivity rate was 2.3% among patients with diabetes, lower than the previously reported rate of 5.7%, likely due to most HCV cases having been detected by prior testing during routine care [26].

Redundant screening remains an issue. Prior studies report that 15.6% of patients undergo repeated anti-HCV testing, and 27.6% of seropositive patients have already received viremia testing [33]. In our cohort, 17.9% of seropositive individuals had received testing and antiviral therapy elsewhere. This may explain our lower HCV viremia rate (37.4%). Developing a real-time, county-wide data-sharing platform for screening data could minimize unnecessary testing and improve care coordination.

Successful implementation of EMR-based screening requires collaboration among multiple stakeholders, including primary care physicians, IT support, laboratory services, and hospital leadership. A top-down approach, incorporating physician education, IT responsiveness, and administrative commitment, played an essential role in the program’s success. Although EMR systems facilitate clinical care, excessive alerts can disrupt usual workflows. We, therefore, limited the pop-up alerting function to five months to avoid fatigue, as most patients with chronic diseases in Taiwan have follow-ups every three months. After this period, the status of viral hepatitis screening was displayed on the computer screen; however, the action for HCV screening was left to the physician’s discretion.

Limitations of this study include the non-mandatory nature of alerts, which may have contributed to some missed screening opportunities. We did not collect non-responding reasons for the physician’s or patient’s non-participation. Additionally, detailed patient comorbidity data were not analyzed. Our single-center design may limit generalizability. Future multicenter studies are needed to validate these findings. Reflex HCV RNA testing was only available for patients screened through health check-ups, resulting in a diagnostic gap for RNA testing. Future programs should integrate reflex testing across all patient pathways. Finally, we did not conduct a cost-effectiveness analysis, although existing literature supports the economic benefit of EMR-based HCV screening in the birth cohort.

## Conclusions

EMR alert systems represent an effective tool to increase HCV screening in a large hospital within a short period. Our study is the first to highlight inter-specialty differences in the implementation within a universal outpatient HCV screening program. The default ordering test resulted in a higher completion rate than physician referrals to health check-ups. Variability was observed in physician engagement, patient willingness, and test completion across different specialties. Continued education and institutional incentives are essential for enhancing HCV screening efforts across all specialties. With appropriate program design, limited alert duration, and continuous stakeholder engagement in the interventions, large-scale identification of unrecognized HCV infections can be achieved. These strategies may be adopted by other institutions worldwide to support in-hospital HCV elimination.

## Supporting information

S1 Data Response to EMR and HCV testing in different specialists. (XLSX)

## Abbreviations:

HCV: Hepatitis C virus
EMR: Electronic medical record
DAA: Direct-acting antivirals
WHO: World Health Organization
SVR: Sustained virological response
DM: Diabetes mellitus
CKD: Chronic kidney disease
ESRD: End-stage renal disease
IT: Information Technology
